# Suvorexant, an FDA-approved dual orexin receptor antagonist, reduces oxycodone self-administration and conditioned reinstatement in male and female rats

**DOI:** 10.3389/fphar.2023.1127735

**Published:** 2023-04-27

**Authors:** Jessica M. Illenberger, Francisco J. Flores-Ramirez, Alessandra Matzeu, Barbara J. Mason, Rémi Martin-Fardon

**Affiliations:** Department of Molecular Medicine, The Scripps Research Institute, La Jolla, CA, United States

**Keywords:** prescription opioid use disorder, reinstatement, dual orexin receptor antagonists, oxycodone, suvorexant

## Abstract

**Background:** The Department of Health and Human Services reports that prescription pain reliever (e.g., oxycodone) misuse was initiated by 4,400 Americans each day in 2019. Amid the opioid crisis, effective strategies to prevent and treat prescription opioid use disorder (OUD) are pressing. In preclinical models, the orexin system is recruited by drugs of abuse, and blockade of orexin receptors (OX receptors) prevents drug-seeking behavior. The present study sought to determine whether repurposing suvorexant (SUV), a dual OX receptor antagonist marketed for the treatment of insomnia, can treat two features of prescription OUD: exaggerated consumption and relapse.

**Methods:** Male and female Wistar rats were trained to self-administer oxycodone (0.15 mg/kg, i. v., 8 h/day) in the presence of a contextual/discriminative stimulus (S^D^) and the ability of SUV (0–20 mg/kg, p. o.) to decrease oxycodone self-administration was tested. After self-administration testing, the rats underwent extinction training, after which we tested the ability of SUV (0 and 20 mg/kg, p. o.) to prevent reinstatement of oxycodone seeking elicited by the S^D^.

**Results:** The rats acquired oxycodone self-administration and intake was correlated with the signs of physical opioid withdrawal. Additionally, females self-administered approximately twice as much oxycodone as males. Although SUV had no overall effect on oxycodone self-administration, scrutiny of the 8-h time-course revealed that 20 mg/kg SUV decreased oxycodone self-administration during the first hour in males and females. The oxycodone S^D^ elicited strong reinstatement of oxycodone-seeking behavior that was significantly more robust in females. Suvorexant blocked oxycodone seeking in males and reduced it in females.

**Conclusions:** These results support the targeting of OX receptors for the treatment for prescription OUD and repurposing SUV as pharmacotherapy for OUD.

## 1 Introduction

Moderate to severe pain management in the United States largely relies on prescription opioids, such as oxycodone, with over 142,000,000 total prescription opioids dispensed in 2020 (Centers for Disease Control and Prevention, 2021). Strikingly, 18.6% of drug overdose fatalities in the same year involved a prescription opioid (Centers for Disease Control and Prevention, 2022). Oxycodone is one of the most prescribed opioids (Wide-Ranging Online Data for Epidemiologic Research, 2021) and, despite similar half-lives (compared to hydrocodone, morphine) or reduced potency (compared to fentanyl, buprenorphine) compared to other prescription opioids, oxycodone ranked highly in abuse potential by patients with opioid use disorder (OUD; Zacny and Lichtor, 2008; Butler et al., 2010; Stoops et al., 2010; Remillard et al., 2019; Kibaly et al., 2021; Vearrier and Grundmann, 2021). Even the appropriate use of prescription opioids can lead to physical dependence and the escalation of drug consumption, contributing to the development of drug misuse and addiction (Volkow et al., 2019). Neuroadaptations that occur in response to repeated drug use contribute to the reinstatement of drug seeking and last well into drug abstinence (Volkow et al., 2019; Koob, 2021). Collectively, these data demonstrate the urgency to develop new therapies to reduce the motivation to seek opioids both before and after periods of abstinence.

The orexin (OX) system, also known as the hypocretin system, is involved in various physiological processes, such as sleep/wake regulation (Chemelli et al., 1999; Hoyer and Jacobson, 2013; Inutsuka and Yamanaka, 2013; Krystal et al., 2013), stress (Berridge et al., 2010; Sargin, 2019), feeding (Sakurai et al., 1998), and reward processing (Harris et al., 2005; Martin-Fardon & Weiss, 2012; Matzeu and Martin-Fardon, 2020). The OX system is strongly recruited by drug use and was shown to incur neuroadaptations with repeated drug use, including cocaine (Zhang et al., 2007; James et al., 2019; Matzeu and Martin-Fardon, 2021), alcohol (Amodeo et al., 2020), nicotine (Kane et al., 2000), and opioids (Georgescu et al., 2003; Thannickal et al., 2018; Fragale et al., 2020; Sadat-Shirazi et al., 2020). Additionally, pharmacological manipulations of OX transmission have been shown to influence drug intake and seeking of alcohol (Moorman and Aston-Jones, 2009; Shoblock et al., 2011; Martin-Fardon & Weiss, 2012), cocaine (Borgland et al., 2006; Martin-Fardon and Weiss, 2014a), and opioids (Smith and Aston-Jones, 2012; Matzeu and Martin-Fardon, 2020). Opioid exposure reportedly increases OX cell counts (Thannickal et al., 2018), and OX cells are activated in the presence of drug-paired stimuli (Harris et al., 2005; Dayas et al., 2008; Jupp et al., 2011; Martin-Fardon et al., 2018). The National Institute on Drug Abuse named OX receptor antagonists and negative allosteric modulators as pharmacotherapies with a high potential of Food and Drug Administration (FDA) approval for the treatment of some aspects of OUD (Rasmussen et al., 2018).

Suvorexant (SUV) is a dual OX receptor antagonist (DORA) approved by the FDA in 2014 and marketed by Merck as Belsomra^®^ for the treatment of insomnia. Suvorexant is highly brain penetrant and has strong and equivalent affinity for both the OX_1_ receptor and OX_2_ receptor in both humans (OX_1_ receptor, K_i_ = 0.55 nM; OX_2_ receptor, K_i_ = 0.35 nM) and rats (OX_1_ receptor, K_i_ = 0.54 nM; OX_2_ receptor, K_i_ = 0.57 nM; Winrow et al., 2011). Acute oral (p. o.) administration of SUV (10–100 mg) in healthy men yielded maximal plasma concentrations 3 h post-administration (Sun et al., 2013). In rats, p.o. administration of 10 mg/kg SUV yielded maximal plasma concentrations 0.5 h after administration (Cox et al., 2010). Alternative DORAs (i.e., almorexant and DORA-1) have been reported to reduce alcohol intake (Anderson et al., 2014; Srinivasan et al., 2012), interfere with the expression of cocaine- and amphetamine-induced conditioned place preference (CPP; Steiner et al., 2013), reduce nicotine conditioned reinstatement (Winrow et al., 2010), and attenuate morphine-induced locomotor sensitization (Steiner et al., 2013), although these compounds have yet to obtain FDA-approval. The acute administration of SUV, however, reduced stimulant (Simmons et al., 2017; Gentile et al., 2018) and alcohol intake in rats (Flores-Ramirez et al., 2022) and reduced opioid withdrawal and craving in patients who were undergoing buprenorphine taper (Huhn et al., 2022). Therefore, SUV may indirectly reduce the risk to opioid relapse by reducing drug craving (primarily via OX_1_ receptor) and normalizing sleep disturbances (primarily via OX_2_ receptor) commonly observed in OUD patients (Peles et al., 2006; Hartwell et al., 2014).

The present study first investigated the ability of SUV to reduce excessive oxycodone intake, utilizing a model of extended oxycodone self-administration access in male and female rats. Effectively treating OUD will require strategies that not only prevent excessive opioid consumption, but also resolve a central problem in treating OUD that is chronic relapsing to opioid use, even after extended periods of abstinence (Rudd et al., 2016; Gostin et al., 2017; Volkow and Collins, 2017). Relapse is often precipitated by environmental stimuli that had repeatedly been paired with drug consumption and therefore acquired incentive-motivational value through associative learning processes. Such environmental stimuli cause relapse by eliciting the expectation of drug availability and drug craving during abstinence (Miller and Gold, 1994; O’Brien et al., 1998; Tiffany and Carter, 1998; Tiffany and Conklin, 2000; van de Laar et al., 2004). Therefore, another goal of this study was to investigate if the blockade of OX receptors with SUV prevents oxycodone-seeking behavior in a conditioned reinstatement model.

## 2 Materials and methods

### 2.1 Animals

Male (*n* = 16) and female (*n* = 16) Wistar rats (Charles River, Wilmington, MA, United States of America), 40–45 days old upon arrival, were housed two per cage in a temperature- and humidity-controlled vivarium on a reverse 12 h/12 h light/dark cycle with *ad libitum* access to food and water. All operant behavior procedures (self-administration, extinction, and conditioned reinstatement sessions) were performed during the rats’ active (dark) phase. These procedures were conducted in strict adherence to the National Institutes of Health’s Guide for the Care and Use of Laboratory Animals and were approved by the Institutional Animal Care and Use Committee of The Scripps Research Institute.

### 2.2 Drugs

Suvorexant (Belsomra^®^; Merck, Whitehouse Station, NJ, United States of America) pills (20 mg) were crushed and dissolved in a 20% vitamin E TPGS (D-α-tocopherol polyethylene glycol 1,000 succinate; Mazuri, Richmond, IN, United States of America) solution (Cox et al., 2010; Guo et al., 2013; Ehlers et al., 2020) which was also used for control (i.e., 0 mg/kg SUV) group injections. Once homogenized, to maximize bioavailability of the compound, SUV was administered 30 min before the test sessions at doses of 0, 10, and 20 mg/kg (5 mL/kg, p. o.; based on previously reported doses: Simmons et al., 2017; Gentile et al., 2018). Oxycodone hydrochloride (Spectrum Chemicals, St. Louis, MO, United States of America) dissolved in 0.9% sodium chloride (Hospira, United States of America) was administered intravenously (i.v., 0.15 mg/kg/0.1 mL; Wade et al., 2014; Matzeu and Martin-Fardon, 2020).

### 2.3 Behavioral testing and procedures

Male and female rats were surgically implanted with jugular catheters while under general anesthesia (isoflurane, 5% for induction, 1%–3% for maintenance). Intravenous catheters made of Micro-Renathane tubing (0.037-inch diameter; Braintree Scientific, Braintree, MA, United States of America) were attached to a guide cannula (Plastics One, Roanoke, VA, United States of America) and secured with dental acrylic cement to an anchoring 2 cm circular mesh. As previously described (Caine and Koob, 1993), the tubing was aseptically inserted and secured in the right jugular vein, and the attached catheter port was placed on the back, closed with a plastic cap and covered with a metal cap to keep the catheter clean and protected. After 7–10 days of recovery, oxycodone self-administration training took place in daily 8-h (extended access) sessions initiated by the extension of two retractable levers into operant chambers (29 cm × 24 cm × 19.5 cm; Med-Associates, St. Albans, VT, United States of America) and presence of the contextual/discriminative stimulus (S^D^; constant 70 dB white noise). Responses on the right “active lever” were reinforced on a fixed-ratio 1 (FR1) schedule by infusion of oxycodone (0.15 mg/0.1 mL/infusion, i. v.) over 4 s, followed by a 20-s timeout period (TO20) signaled by the illumination of a cue light above the lever. Responses on the left inactive lever were recorded but had no scheduled consequences.

**FIGURE 1:**
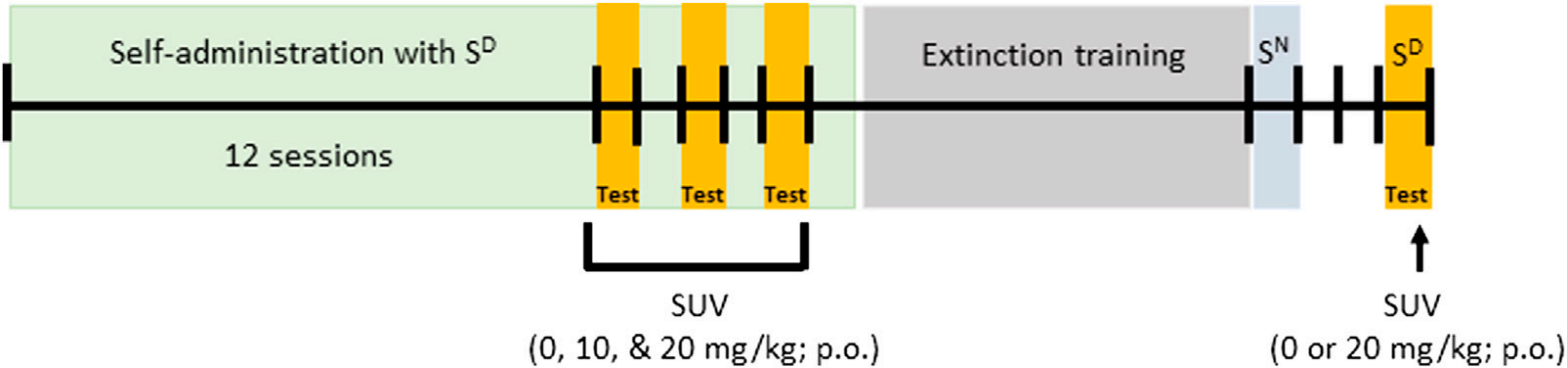
Experimental design.

Testing procedures were based on our previously published methods (Matzeu and Martin-Fardon, 2020) and are represented in Figure 1. Self-administration training sessions were conducted 5 days/week (Monday to Friday), interspersed by 48 h of withdrawal when the rats were kept in their home cage (Saturday and Sunday). The rats were scored for spontaneous physical withdrawal signs (i.e., jumps, paw tremors, teeth chattering, wet dog shakes, piloerection, and ptosis; Schulteis et al., 1999; Rehni et al., 2013; Matzeu and Martin-Fardon, 2020) once weekly (after sessions 1, 7, 14, and 18), 1 h before the self-administration session that corresponded to a 15-h abstinence period. The following severity scale: 0 = no signs, 1 = moderate, 2 = severe, was used to score each withdrawal measure and the sum was used as a quantitative measure of withdrawal severity. Once oxycodone self-administration was acquired and dependence was confirmed, 0, 10, or 20 mg/kg SUV (p.o.) was administered 30 min before the start of oxycodone self-administration. The SUV doses were tested in a within-subjects, Latin-square design every other day (sessions 13, 15, and 17) to control for possible order effects of SUV dosing. Self-administration sessions without prior dosing were carried out on alternate days (sessions 14, 16, and 18) to prevent carry-over effects or SUV-induced extinction of active lever pressing.

After the completion of testing the effects of SUV on oxycodone self-administration, responses on the active lever were extinguished in daily 1-h extinction sessions, during which responses had no programmed consequences (i.e., no oxycodone and no cue presentation) until rats reached an average of ≤10 active lever responses (Figure 1). To control for specificity of the S^D^ to reinstate extinguished oxycodone seeking, a neutral stimulus (S^N^; i.e., the illumination of a 2.8 W house light at the top of the chamber’s front panel) was presented in place of the S^D^ during a 1-h session during which, responding on the right active lever was followed by a TO20 signaled by a 70 dB (70 kHz) intermittent beeping tone. After the S^N^ session, the rats were returned to their regular home cages. Two days after S^N^ testing, 1 h prior to testing conditioned reinstatement of oxycodone-seeking behavior, the rats were scored for spontaneous physical withdrawal signs. The rats were then administered with 0 or 20 mg/kg SUV (between-subjects), and 30 min later were presented with the S^D^ in a 1-h conditioned reinstatement session, and tested for oxycodone-seeking behavior (Figure 1).

### 2.4 Effect of SUV on sweetened condensed milk-reinforced behavior

To control for sedative behavioral effects of SUV (i.e., non-specific inhibitory behavioral effects), an additional group of male (*n* = 6) and female (*n* = 6) rats were trained to orally self-administer sweetened condensed milk (SCM; diluted 2:1 v/v, 0.1 mL; e.g., Martin-Fardon and Weiss, 2014a; Martin-Fardon and Weiss, 2014b; Matzeu et al., 2017; Matzeu et al., 2018) during 1-h daily (5 days/week) sessions on an FR1 TO20 (signaled by the illumination of a cue light above the right active lever) schedule for 6 sessions. The SCM group did not undergo surgery. Once SCM self-administration was acquired, 0 or 20 mg/kg SUV (p.o.) was administered 30 min before the start of SCM self-administration. To prevent carry-over effects or SUV-induced extinction of active lever pressing, a SCM self-administration session without prior dosing was carried out before testing the next dose. The order in which animals received each dose of SUV was randomized.

### 2.5 Statistical analyses

Statistical analyses on the development and expression of oxycodone dependence were conducted separately in males and females. However, unpaired *t*-test were used to confirm consistency with the literature that 1) females consume more oxycodone than males and 2) females reinstate to a higher level than males (Fulenwider et al., 2020; Kimbrough et al., 2020; Vazquez et al., 2020; Zanni et al., 2020; Towers et al., 2022). The acquisition of oxycodone and SCM self-administration was analyzed using two-way repeated-measures analysis of variance (RMANOVA), with session and lever (active vs. inactive) as factors. Oxycodone withdrawal scores were analyzed using the non-parametric Friedman test and Wilcoxon matched-pairs signed rank test. Non-parametric Spearman’s correlations determined if withdrawal scores and the number of oxycodone infusions earned in sessions 1, 7, 14, and 18 were significantly correlated. The effects of SUV on oxycodone self-administration and conditioned reinstatement were analyzed using two-way RMANOVAs with dose (self-administration) or session (extinction, S^N^, and S^D^ for conditioned reinstatement) and lever as factors. Follow-up interrogation of the time-course of 8-h oxycodone self-administration following SUV administration were conducted with RMANOVAs. The effect of SUV on active and inactive responses during SCM self-administration was analyzed using two-way RMANOVAs. Significant effects in the omnibus ANOVAs were followed by Bonferroni *post hoc* tests. For withdrawal scores, Dunn’s *post hoc* tests were used. The statistical analyses were performed using GraphPad Prism 9.3.1 software. All data are expressed as the mean ± SEM. Values of *p* < 0.05 were considered statistically significant.

## 3 Results

*A priori* power analyses were conducted using G*Power 3 (Faul et al., 2007) to ensure enough animals were included to test the interactions between two within-subjects’ factors or two mixed factors using two-tailed tests. An estimated effect size of 0.5 was used based on data from previous studies in our laboratory. Results showed that a sample of 6–12 rats per sex were required to achieve a power of 0.80.

Three rats were lost during the acquisition of oxycodone self-administration (two because of catheter failure and one because of health complications), therefore, 15 males and 14 females were tested on the effect of SUV on oxycodone self-administration. For oxycodone conditioned reinstatement, all the males were tested (i.e., *n* = 8 at the 0-mg dose and *n* = 7 at the 20-mg/kg dose). However, due to the restricted amount of SUV available at the time of testing, 10 out the 14 females were tested for conditioned reinstatement (i.e., *n* = 6 at the 0-mg/kg dose and *n* = 4 at the 20-mg/kg dose).

**FIGURE 2:**
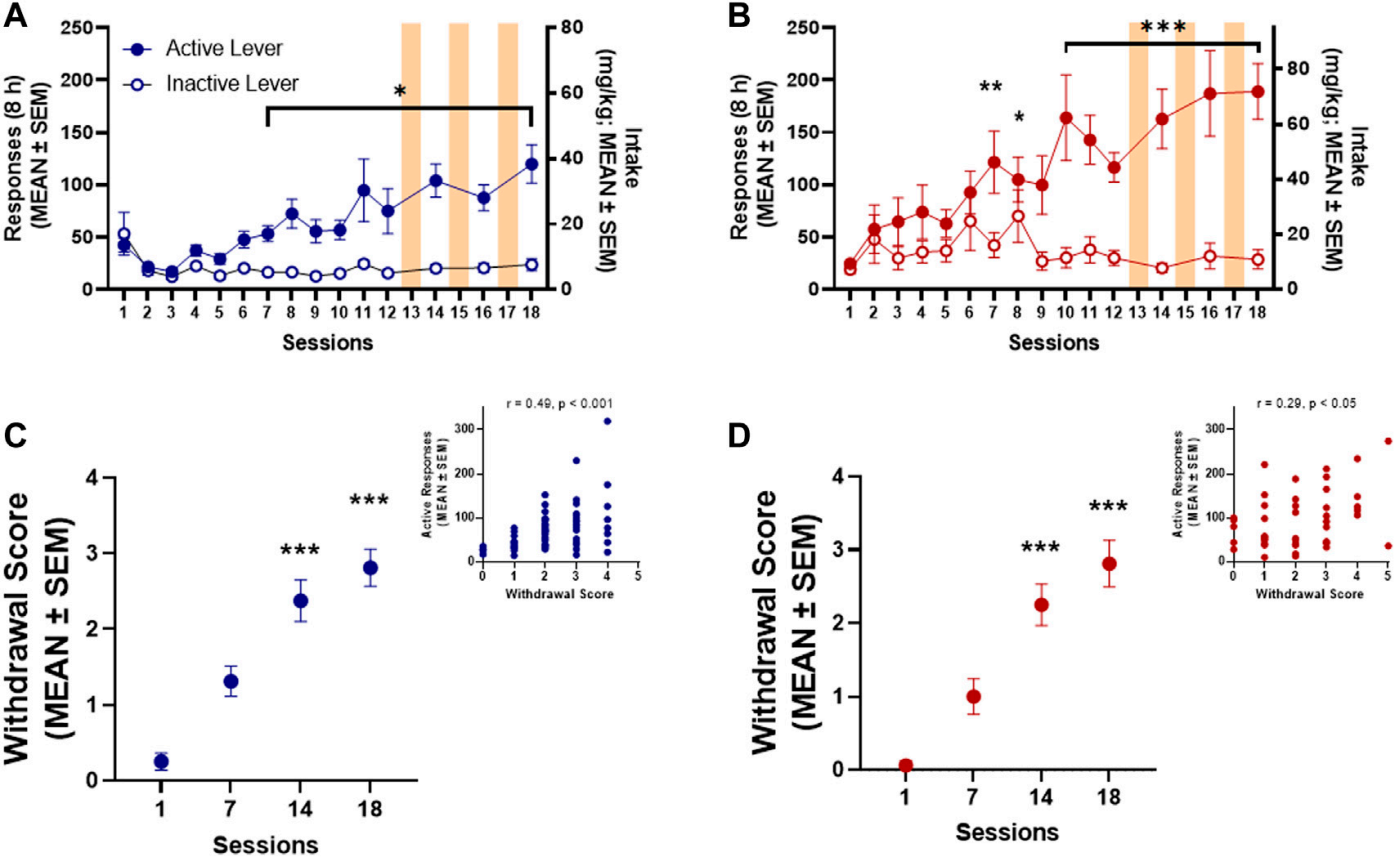
Oxycodone self-administration in males (*n* = 15) **(A)** and females (*n* = 14) **(B)**. *p < 0.05, **p < 0.01, ***p < 0.001, vs. inactive lever (Dunnett’s multiple-comparison post hoc test). Somatic withdrawal in both males **(C)** and females **(D)**. ***p < 0.001, vs. session 1 (Dunn’s multiple-comparison post hoc test). Insets: Correlation plot between somatic withdrawal score and number of oxycodone infusions.

Both male (*n* = 15) and female (*n* = 14) rats acquired oxycodone self-administration over the 12 sessions of training (Figures 2A, B). Responding on the active lever was significantly higher than responding on the inactive lever by session 7 in both male (*p* = 0.031, Bonferroni *post hoc* test following two-way RMANOVA, session × lever: *F*_14,196_ = 7.09, *p* ≤ 0.0001; Figure 2A) and female (*p* = 0.004, Bonferroni *post hoc* test following two-way RMANOVA, session × lever: *F*_14,182_ = 6.579, *p* ≤ 0.0001; Figure 2B) rats. At the end of the 12 training sessions, females self-administered significantly more oxycodone than males (48.55 ± 5.01 mg/kg vs. 22.49 ± 4.83 mg/kg, respectively; unpaired *t*-test, *t*_27_ = 3.75, *p* = 0.001). The number of responses on the active lever remained stable and significantly higher than responses emitted on the inactive lever in sessions 14, 16, and 18 after SUV testing (i.e., sessions 13, 15, and 18) in both males (*p* ≤ 0.0001, Bonferroni *post hoc* test following two-way RMANOVA; Figure 2A) and females (*p* ≤ 0.0001, Bonferroni *post hoc* test following two-way RMANOVA; Figure 2B). Somatic withdrawal (Figures 2C,D) was significantly more pronounced by session 14 of oxycodone self-administration vs. the first measurement in both males (*p* ≤ 0.0001, vs. session 1, Dunn’s test following Friedman test: 36.23, *p* ≤ 0.0001; Figure 2C) and females (*p* = 0.001, vs. session 1, Dunn’s test following Friedman test: 31.76, *p* ≤ 0.0001; Figure 2D). The number of active lever responses during oxycodone self-administration sessions significantly correlated with somatic signs of withdrawal that were measured at 15 h of abstinence in males (Spearman *R* = 0.49, *p* ≤ 0.0001; Figure 2C **inset**) and females (Spearman *R* = 0.29, *p* = 0.048; Figure 2D **inset**).

**FIGURE 3:**
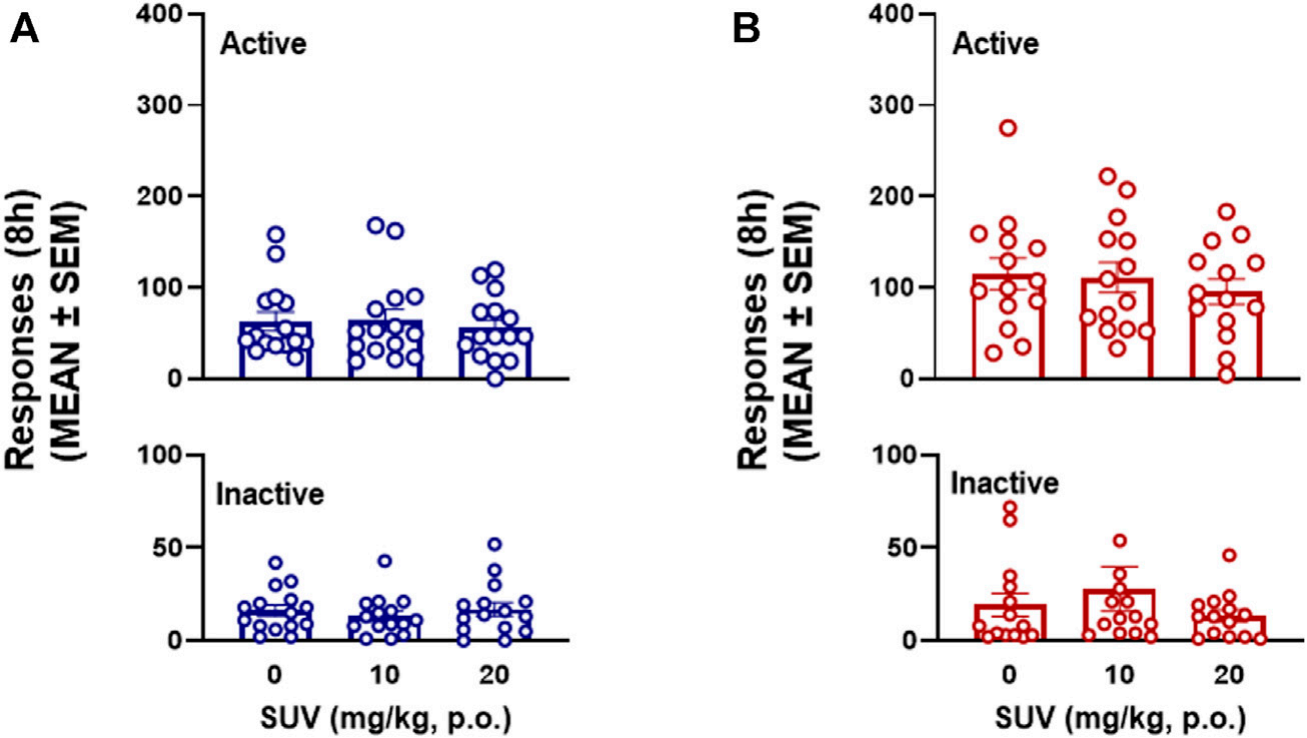
Effect of suvorexant on 8-h oxycodone self-administration in males (*n* = 15) **(A)** and females (*n* = 14) **(B)**.

**FIGURE 4:**
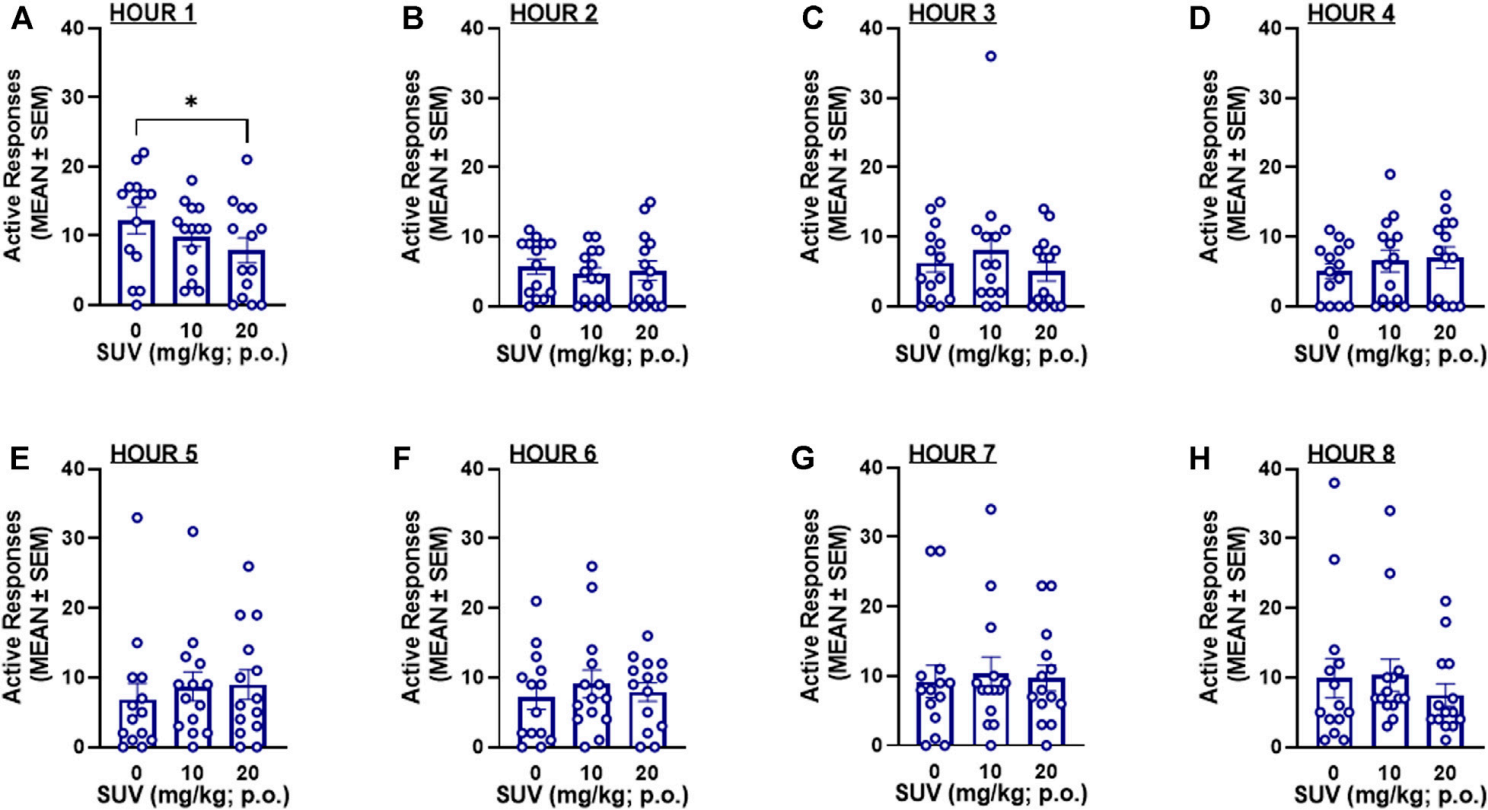
Oxycodone self-administration following suvorexant administration in males (*n* = 15) during the first hour of the session **(A)** and during each subsequent hour **(B)**: hour 2, **(C)** hour 3, **(D)** hour 4, **(E)** hour 5, **(F)** hour 6, **(G)** hour 7, **(H)** hour 8 of the 8-h session. *p < 0.05, vs. 0 mg/kg (Bonferroni multiple-comparison *post hoc* test).

**FIGURE 5:**
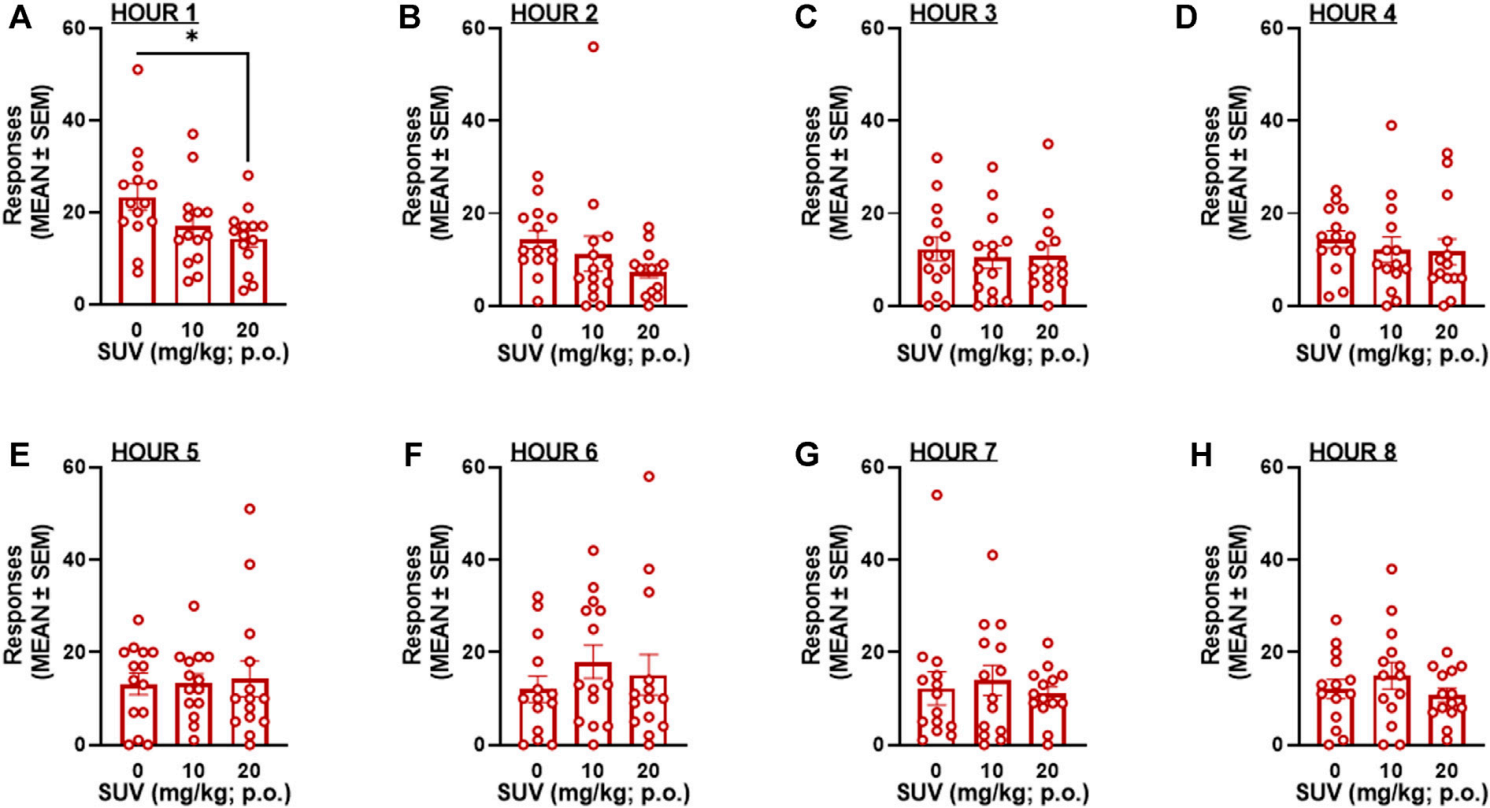
Oxycodone self-administration following suvorexant administration in females (*n* = 14) during the first hour of the session **(A)** and during each subsequent hour **(B)**: hour 2, **(C)** hour 3, **(D)** hour 4, **(E)** hour 5, **(F)** hour 6, **(G)** hour 7, **(H)** hour 8 of the 8-h session. *p < 0.05, vs. 0 mg/kg (Bonferroni multiple-comparison *post hoc* test).

The total number of responses on active and inactive levers over the 8-h self-administration session was not modified by SUV in males (two-way RMANOVA, dose: *F*_2,28_ = 0.217, *p* = 0.806; *F*_1,14_ = 26.07, lever: *p* = 0.0002; dose × lever: *F*_2,28_ = 0.777, *p* = 0.469; Figure 3A) or females (two-way RMANOVA, dose: *F*_2,26_ = 1.545, *p* = 0.232; lever: *F*_1,13_ = 74.80, *p* ≤ 0.0001; dose × lever: *F*_2,26_ = 0.672, Figure 3B). Because maximal plasma concentrations of SUV are reported to occur within the first 0.5 h of administration (Cox et al., 2010), the number of responses within each hour of the 8-h session was analyzed by RMANOVAs. At the highest dose tested (20 mg/kg), SUV decreased oxycodone self-administration during the first hour in males (*p* = 0.012, vs. 0 mg/kg, Bonferroni *post hoc* test following RMANOVA: *F*_2,26_ = 5.05, *p* = 0.014; Figure 4A) and females (*p* = 0.018, vs. 0 mg/kg, Bonferroni *post hoc* test following RMANOVA: *F*_2,26_ = 4.77, *p* = 0.017; Figure 5A), without any effect during the remaining hours (hours 2–8) in males (RMANOVA: *p* > 0.05; Figures 4B–H) or females (RMANOVA: *p* > 0.05; Figures 5B–H) or inactive lever responding during any of the 8-h sessions (RMANOVA: *p* > 0.05).

At the initiation of extinction training (i.e., session 1), males emitted an average of 22.87 ± 6.08 responses on the active lever vs. 56.93 ± 10.19 responses for females (unpaired *t*-test, *t*_27_ = 2.92, *p* = 0.007). Males took an average of 4.53 ± 0.83 sessions to reach extinction (i.e., ≤10 responses at the active lever). Females took significantly more sessions (9.57 ± 1.34) to reach extinction than males (unpaired *t*-test, *t*_27_ = 3.24, *p* = 0.003).

**FIGURE 6:**
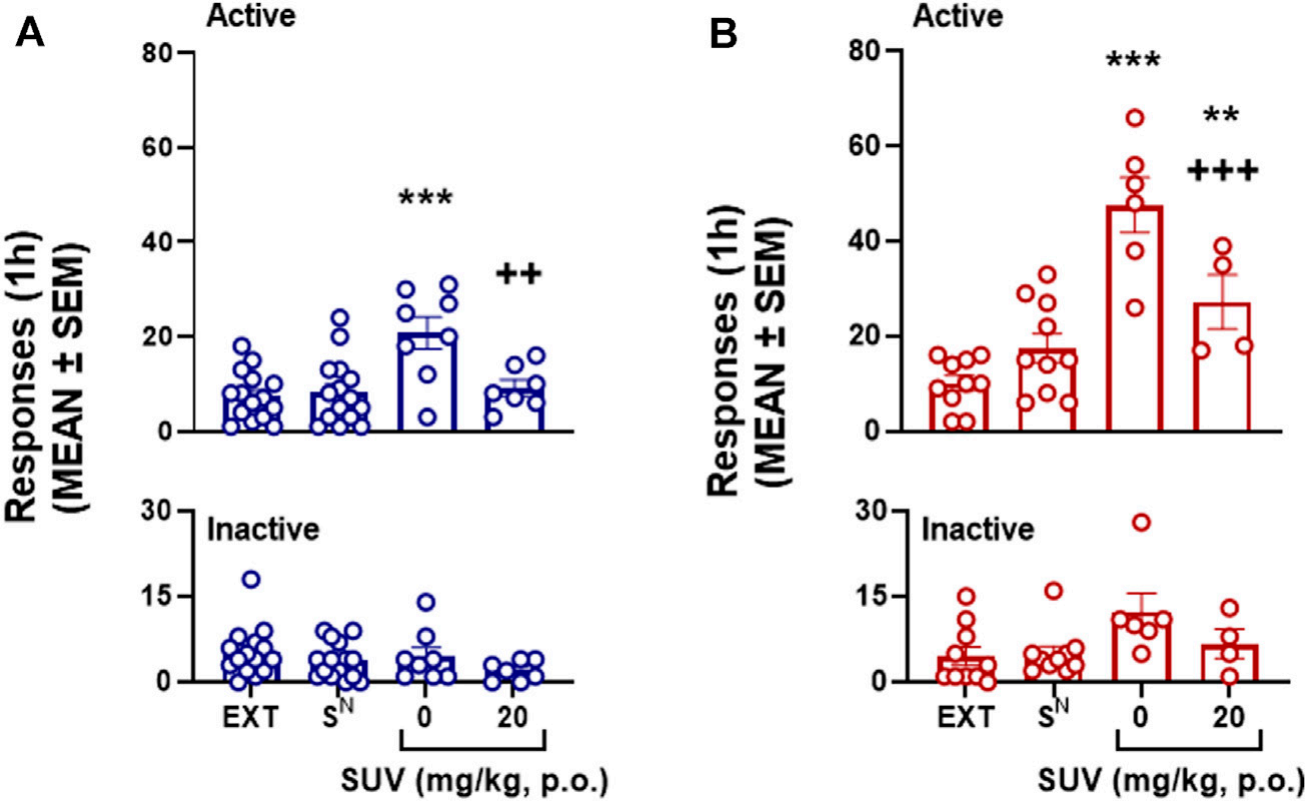
Effect of suvorexant on conditioned reinstatement of oxycodone-seeking behavior in males (*n* = 15, *n* = 7-8 per dose) **(A)** and females (*n* = 10, *n* = 4-6 per dose) **(B)**. **p < 0.01, ***p < 0.001, vs. respective EXT; ^++^ *p* < 0.01, ^+++^ *p* < 0.001, vs. respective 0 mg/kg (Bonferroni multiple-comparison *post hoc* test). EXT, extinction.

Following extinction training, males (Wilcoxon matched-pairs signed rank test: 105, *p* ≤ 0.0001) and females (Wilcoxon matched-pairs signed rank test: 88, *p* = 0.0007) exhibited a significant decrease in physical withdrawal signs compared to that displayed on self-administration session 18. Presentation of the S^D^ (but not the S^N^) under vehicle conditions (i.e., 0 mg/kg SUV) elicited the reinstatement of oxycodone seeking in males (*p* = 0.0003, 0 mg/kg vs. EXT, Bonferroni *post hoc* test following two-way mixed-effects analysis: session × lever: *F*_3,35_ = 6.72, *p* = 0.0003; Figure 6A) and females (*p* ≤ 0.0001, 0 mg/kg vs. EXT, Bonferroni *post hoc* test following two-way mixed-effects analysis: session × lever: *F*_3,22_ = 15.01, *p* ≤ 0.0001; Figure 6B). Females exhibited stronger reinstatement than males (unpaired *t*-test: *t*_12_ = 4.27, *p* = 0.001; Figures 6A,B for comparison). The 20 mg/kg dose of SUV blocked conditioned reinstatement in males (*p* = 0.01, vs. 0 mg/kg, Bonferroni *post hoc* test; Figure 6A), to extinction levels (*p* ≥ 0.999, vs. EXT, Bonferroni *post hoc* test; Figure 6A). In females, although 20 mg/kg SUV significantly reduced conditioned reinstatement (*p* = 0.019, 0 mg/kg vs. 20 mg/kg, Bonferroni *post hoc* test; Figure 6B), substantially significant reinstatement remained (*p* = 0.038, vs. EXT, Bonferroni *post hoc* test; Figure 6B). Inactive responses were not significantly affected by any of the tests (*p* > 0.05, Bonferroni *post hoc* tests).

**FIGURE 7:**
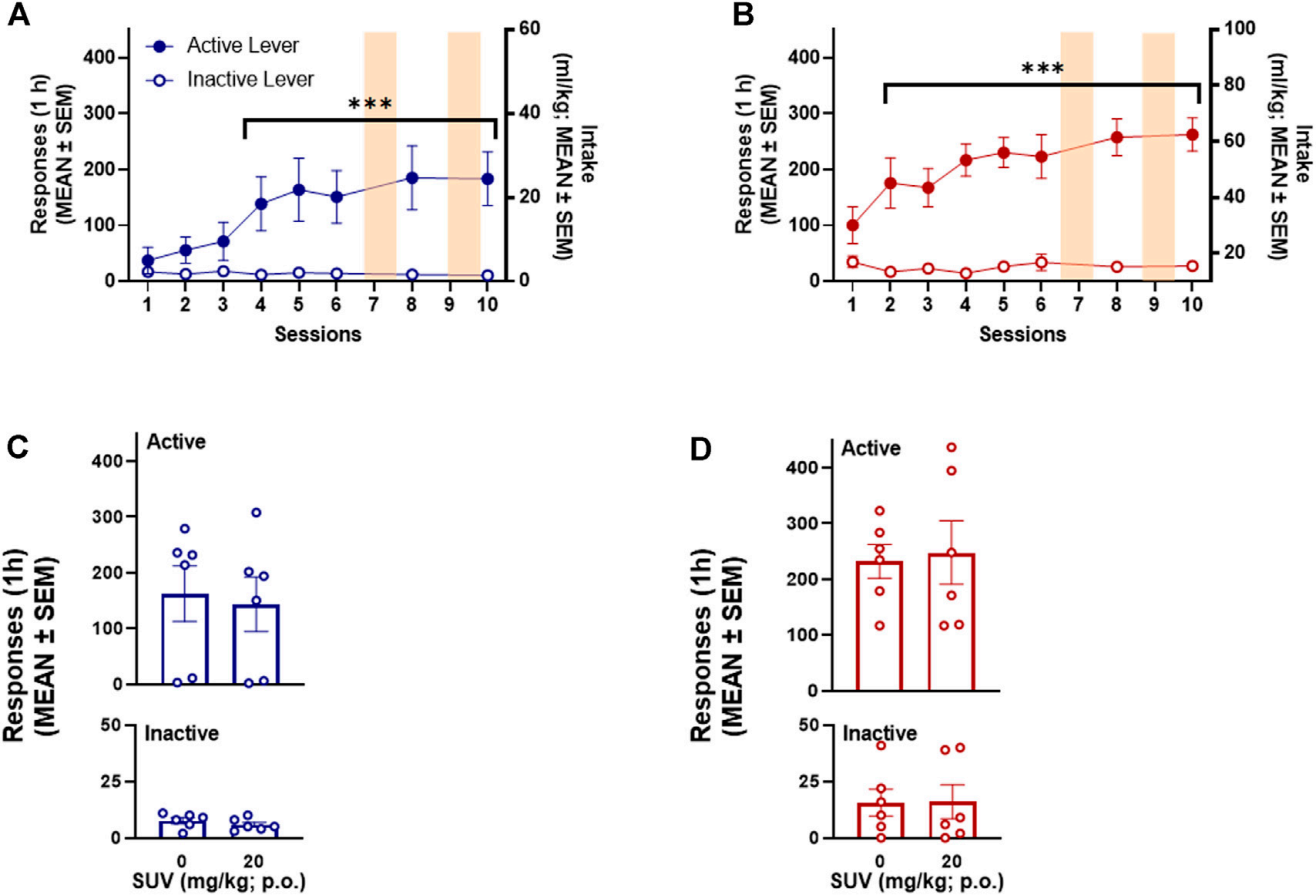
Effect of suvorexant on SCM-reinforced behavior. SCM self-administration in males (*n* = 6) **(A)** and females (*n* = 6) **(B)**. ***p < 0.001, vs. inactive lever (Dunnett’s multiple-comparison *post hoc* test). Lack of effect of SUV on active and inactive responses during 1-h SCM self-administration in males (*n* = 6) **(C)** and females (*n* = 6) **(D)**.

Over the 6 training sessions, both male (*n* = 6) and female (*n* = 6) rats acquired SCM self-administration (Figures 7A, B). Responding on the active lever was significantly higher that responses on the inactive lever by session 4 in males (*p* = 0.028, Bonferroni *post hoc* test following two-way RMANOVA, session × lever: *F*_6,30_ = 4.36, *p* = 0.0008; Figure 7A) and session 2 in females (*p* ≤ 0.0001, Bonferroni *post hoc* test following two-way RMANOVA, session × lever: *F*_6,30_ = 11.09, *p* = 0.0004; Figure 7B). At the end of the 6 training sessions, females self-administered significantly more SCM than males (61.67 ± 5.72 mg/kg vs. 29.31 ± 8.35 mg/kg, respectively; unpaired *t*-test, *t*_10_ = 3.20, *p* = 0.009). The number of responses on the active lever remained significantly higher than responses on the inactive lever during the baseline session between SUV testing (i.e., session 10) in both males (*p* ≤ 0.0001, Bonferroni *post hoc* test following two-way RMANOVA; Figure 7A) and females (*p* ≤ 0.0001, Bonferroni *post hoc* test following two-way RMANOVA; Figure 7B). The total number of responses on active and inactive levers over the 1-h SCM self-administration session was not modified by SUV in males (two-way RMANOVA: dose: *F*_1,5_ = 1.520, *p* = 0.272; lever: *F*_1,5_ = 8.76, *p* = 0.031; dose × lever: *F*_1,5_ = 1.203, *p* = 0.322, Figure 7C) or females (two-way RMANOVA: dose: *F*_1,5_ = 0.119, *p* = 0.743; lever: *F*_1,5_ = 43.66, *p* = 0.001; dose × lever: *F*_1,5_ = 0.139, *p* = 0.724; Figure 7D).

## 4 Discussion

In the present study, both male and female rats readily acquired oxycodone self-administration with 8-h access, confirming earlier findings (e.g., Wade et al., 2014; Mavrikaki et al., 2017; Nguyen et al., 2018; 2019; Blackwood et al., 2019; Matzeu and Martin-Fardon, 2020; Kimbrough et al., 2020). Acquisition of oxycodone self-administration was characterized by significant increases in oxycodone intake and was correlated with exhibited physical signs of oxycodone withdrawal, verifying dependence. The present study also found that the administration of SUV, an FDA-approved DORA for the treatment of insomnia, treated two critical aspects of OUD by 1) decreasing oxycodone intake and 2) reducing the reinstatement of oxycodone seeking induced by oxycodone contextual stimuli. Altogether, the results endorse targeting the OX system for the treatment of substance use disorders and highlight the potential significance of repurposing SUV for the treatment of prescription OUD.

Oxycodone self-administration under extended access conditions was described as a reliable model for studying OUD, which was supported herein. Both male and female rats increased their intake of oxycodone over the course of self-administration training and exhibited withdrawal signs that significantly correlated with the amount of oxycodone consumed, verifying dependence. Interestingly, at the end of self-administration training (session 12), females took approximately twice the amount of oxycodone as males (48.55 ± 5.01 mg/kg vs. 22.49 ± 4.83 mg/kg, respectively), consistent with another study that used virtually the same conditions described herein (Kimbrough et al., 2020). Suvorexant significantly decreased oxycodone intake at the highest dose tested (20 mg/kg) in both males and females, an effect that occurred in the first hour of the 8-h session. This effect was not the result of a non-specific locomotion effect of SUV (sedation) because it did not affect SCM-reinforced behavior. This suggests that SUV could be beneficial in reducing excessive intake that is seen during initiation of the self-administration session (i.e., loading phase) but not during later parts of the self-administration session (i.e., maintenance phase). The results are consistent with previous reports that the OX_1_ receptor antagonist SB334867 reduced oxycodone (Matzeu and Martin-Fardon, 2020) and heroin (Smith and Aston-Jones, 2012) self-administration and the motivation for fentanyl (Fragale et al., 2019; Fragale et al., 2020) and remifentanil (Porter-Stransky et al., 2017; Mohammadkhani et al., 2020) on behavioral economics tasks. OX_2_ receptor antagonists have been reported to reduce heroin (Schmeichel et al., 2014) but not oxycodone (Matzeu and Martin-Fardon, 2020) self-administration, suggesting different roles for OX_1_ and OX_2_ receptors in the expression of opioid intake. Likewise, nicotine self-administration is reduced by OX_1_ receptor (Hollander et al., 2008) but not OX_2_ receptor (Uslaner et al., 2014) antagonism. Alcohol self-administration is reduced by both OX_1_ receptor (Lawrence et al., 2006; Richards et al., 2008; Anderson et al., 2014) and OX_2_ receptor (Shoblock et al., 2011; Brown et al., 2013) antagonists. Cocaine self-administration is also reduced by OX_1_ receptor antagonists (Hutcheson et al., 2011; Hollander et al., 2012; Brodnik et al., 2015; Foltin and Evans, 2018), but the salience or level of effort that is required to obtain drug may influence the ability of OX_1_ receptor antagonists to reduce cocaine-seeking behavior (Borgland et al., 2009; España et al., 2010). Still to be tested, however, is whether SUV may also reduce the intake of other prescription opioids with similar or shorter duration of action vs oxycodone (e.g., fentanyl, hydrocodone, morphine, etc.), as SUV effects occurred within the first hour of the self-administration session. Likewise, SUV may be less effective at reducing intake of prescription opioids with longer half-lives (e.g., buprenorphine, methadone, etc.). Nevertheless, the current results add to the existing preclinical literature that SUV can reduce oxycodone intake in addition to stimulants (Simmons et al., 2017; Gentile et al., 2018) and alcohol intake (Flores-Ramirez et al., 2022), supporting that SUV may be an effective treatment strategy for reducing intake of various drugs of abuse (i.e., opioids, stimulants, and alcohol). However, despite preclinical reports indicating that 10 and 30 mg/kg SUV can reduce stimulant intake in rats (Simmons et al., 2017; Gentile et al., 2018), a recent report indicates that 10 and 20 mg/kg SUV increased cocaine intake in a clinical setting (Stoops et al., 2022). It is possible that maintaining SUV levels through chronic (minimum of 3 days) administration (Stoops et al., 2022) elicits different effects on drug intake and seeking compared to that of acute SUV administration utilized in preclinical studies (e.g., current study; Simmons et al., 2017; Gentile et al., 2018; Flores-Ramirez et al., 2022). As such, clinical dose-response studies on the efficacy of SUV to reduce oxycodone intake and seeking under acute and chronic treatment regimens should be fully evaluated.

A secondary objective of the present study was to test whether SUV prevents oxycodone conditioned reinstatement following extinction training. Despite the disappearance of the physical signs of withdrawal at the time of the oxycodone conditioned reinstatement tests, presentation of the oxycodone S^D^ elicited the reinstatement of oxycodone seeking in males and females paralleling what is observed in the clinic where high vulnerability to relapse is still an issue even during post-detoxification period (e.g., Volkow et al., 2016; Koob, 2021). The administration of SUV (20 mg/kg) significantly reduced the conditioned reinstatement of oxycodone-seeking behavior in males and females, with a stronger efficacy in males. These findings provide preclinical evidence of the efficacy of OX receptor blockade in reducing oxycodone-seeking behavior. Consistent with the literature reporting the actions of OX receptor antagonists on drug intake, previous studies showed that the blockade of OX_1_ receptors with SB334867 can prevent conditioned reinstatement of various drugs of abuse (i.e., opioids: Smith and Aston-Jones, 2012; Porter-Stransky et al., 2017; Fragale et al., 2019; Matzeu and Martin-Fardon, 2020; alcohol; Lawrence et al., 2006; Jupp et al., 2011; Martin-Fardon and Weiss, 2014b; nicotine; Plaza- Zabala et al., 2013; and cocaine; Smith et al., 2009; Smith et al., 2010; Hutcheson et al., 2011; Martin-Fardon and Weiss, 2014a; Bentzley and Aston-Jones, 2015). While another DORA, TCS1102, was reportedly unable to significantly decrease the conditioned reinstatement of alcohol- (Brown et al., 2013) or nicotine- (Plaza-Zabala et al., 2013; Khoo et al., 2017) seeking behavior, recent experiments from our group revealed that SUV (5 mg/kg) effectively reduced stress-induced reinstatement of alcohol seeking in alcohol-dependent rats (Flores-Ramirez et al., 2022). It is currently unclear if the differences in TCS1102 and SUV efficacy to reduce alcohol seeking are due to differences in the procedures utilized (i.e., conditioned vs stress-induced reinstatement) or in the potency to inhibit OX_2_ receptors (TCS1102: K_i_ = 3.0; Bergman et al., 2008) (SUV K_i_ = 0.35 nM; Winrow et al., 2011). More so, while the efficacy of each OX receptor antagonist should be compared directly, SUV holds a significant advantage over SB334867 and TCS1102 as it has already been approved by the FDA as exhibiting greater medical utility than risk. Likewise, while other DORAs have also been FDA-approved for the treatment of insomnia (lemborexant and daridorexant), the ability of these drugs to reduce drug intake and seeking have yet to be tested.

Although the influence of sex on the development and expression of oxycodone dependence was not directly assessed in the current study, the results are in agreement with the existing literature indicating that female rats exhibit a higher motivation to obtain oxycodone compared to male rats (Fulenwider et al., 2020; Kimbrough et al., 2020; Zanni et al., 2020). In clinical settings, women reportedly experience greater spontaneous (Back et al., 2011) and cue-induced (Yu et al., 2007; Moran et al., 2018) opioid craving and report more severe complications with drug use (Hernandez-Avila et al., 2004; Huhn et al., 2019). Likewise, female rats exhibit higher extinction responding and enhanced conditioned reinstatement of heroin (Vazquez et al., 2020) and fentanyl (Towers et al., 2022). Furthermore, the current results show that the ability for the oxycodone-paired S^D^ to reinstate oxycodone-seeking behavior was significantly stronger in females under vehicle conditions (50.67 ± 8.19 responses vs. 20.75 ± 3.40 responses in males). Therefore, females may exhibit greater activation of neural circuits that are activated in the presence of opioids and opioid-conditioned cues when compared with males, which could explain the stronger reinstatement of oxycodone-seeking behavior in females. The precise mechanisms responsible for the sex differences described here and in the literature are yet to be elucidated. However, sex-specific changes in gene expression (George et al., 2021) and/or dopaminergic reactivity (Mayberry et al., 2022) with opioid use have recently been reported. Sex hormones may also influence the effects of opioids as opioid receptor availability (Smith et al., 2006) and methadone doses needed for women in OUD treatment (Chiang et al., 2017) have been shown to vary with estrogen and estradiol levels, respectively. Evidence that women are more likely to be prescribed opioids and misuse prescription opioids than men (Hirschtritt et al., 2018) highlights the urgency to elucidate the specific mechanisms that underlie sex differences in the sensitivity to opioid use and dependence.

Even though SUV is approved for the treatment of insomnia, therefore reducing wakefulness, it is unlikely that reductions of oxycodone self-administration and conditioned reinstatement described here were the result of heavy sedation. Reductions in the number of responses were not observed during 1-h SCM self-administration sessions and were specific to the behavior directed at the active lever during oxycodone self-administration and conditioned reinstatement testing. Notably, our laboratory has also reported that alcohol-reinforced behavior in non-dependent animals was not significantly reduced by the same 20 mg/kg dose of SUV as that used in the current studies (Flores-Ramirez et al., 2022). These results are consistent with earlier findings reporting that the effects of pharmacological manipulation of the OX system are more likely to be observed when the OX system is hyperactivated (Mahler et al., 2014; Pich and Melotto, 2014) such as, for example, when studying behaviors directed towards highly salient rewards (Borgland et al., 2009; Cason and Aston-Jones, 2013; Zink et al., 2018). These findings are also consistent with other studies that showed that blockade of OX_1_ receptor or both OX_1_ and OX_2_ receptors selectively effect drug-over food-seeking behavior in rats with a history of drug exposure (Winrow et al., 2010; Hollander et al., 2012; Martin-Fardon and Weiss, 2014a; Martin-Fardon et al., 2018; also see Cason et al., 2010). As such, the results of the current studies and previous reports support that the ability for SUV to reduce oxycodone intake and seeking are not likely to be solely due to sedative effects of SUV.

OX cell bodies are found in the hypothalamus only, but OX neurons project throughout the brain and to regions that are pivotal in the regulation of motivation and responses to drug-related stimuli (Peyron et al., 1998; Fadel and Deutch, 2002; Baldo et al., 2003; Sakurai and Mieda, 2011). Suvorexant may reduce drug intake and drug seeking by acutely attenuating the effects of drug-induced neuroadaptations of the OX system (see Georgescu et al., 2003; Thannickal et al., 2018; Fragale et al., 2020; Sadat-Shirazi et al., 2020) and/or downstream circuits. For example, chronic SUV treatment with higher doses than those utilized in the current studies were shown to block morphine-induced enhancement of whole brain CREB and p-ERK protein expression (Esmaili-Shahzade-Ali-Akbari et al., 2021). Although approaches utilizing systemic administration of OX ligands to understand how the OX system influences motivational processes that underlie compulsive opioid taking and seeking have been successful, it is also important to uncover the unique contributions of discreet brain regions and circuits by correlating neural activities with changes in drug-seeking behavior. Such pharmacological manipulations of specific OX circuits have yet to be conducted in combination with opioid self-administration and conditioned reinstatement models but are outside the scope of the current study. Nevertheless, the existing literature suggests that the actions of OX within numerous brain regions contribute to the expression of OUD. For example, OX receptor activation in the ventral tegmental area enhances drug-induced dopaminergic transmission (see Harris et al., 2005; Baimel and Borgland, 2012) and, as such, the administration of OX receptor antagonists in this region attenuated the acquisition and expression of morphine-induced CPP (Narita et al., 2006; Aston-Jones et al., 2010; Farahimanesh et al., 2017). Likewise, OX receptor signaling in the nucleus accumbens also contributes to the acquisition and extinction of morphine-induced CPP (Sadeghi et al., 2016; Alizamini et al., 2017; Farahimanesh et al., 2018). In the locus coeruleus, OX increases tyrosine hydroxylase expression (McGregor et al., 2022) and stimulation of OX receptors in the locus coeruleus modulates glutamate (Mousavi et al., 2014; Hooshmandi et al., 2017b; Ghaemi-Jandabi et al., 2017) and GABA transmission (Davoudi et al., 2016) shown to contribute to the expression of morphine withdrawal. OX_1_ receptor signaling in the lateral paragigantocellularis (Rezaei et al., 2020) and dorsal hippocampus (Hooshmandi et al., 2017a) has also been associated with the expression of opioid withdrawal. OX receptor signaling in the dorsal hippocampus has also been shown to be involved in the acquisition, expression, and reinstatement of morphine-induced CPP (Guo et al., 2015; Sadeghi et al., 2016; Alizamini et al., 2018; Farahimanesh et al., 2018; also see Zarrabian et al., 2020; Vaseghi et al., 2022). These findings suggest that OX system activities throughout a wide system of brain regions contribute to the expression of behaviors related to opioid taking and seeking. Further testing will be required to delineate the exact anatomical and network bases of the behavioral effects of SUV.

Finally, it should be noted that the doses utilized in the present study (10 and 20 mg/kg) are higher than the doses found to be required to achieve at least 90% OX receptor occupancy (Cox et al., 2010) and to reduce active wake time in drug-naive rats with an OX receptor occupancy ≥65% (Gotter et al., 2013). As such, one may argue that off-target effects (on non-OX receptors) of 20 mg/kg SUV could have contributed to reductions in oxycodone intake and seeking. To note, however, that SUV is reportedly highly selective for OX_1_/OX_2_ receptors antagonism, having ∼6000-fold intrinsic selectivity over 170 known receptors and enzymes (Cox et al., 2010; Winrow et al., 2011). This suggests that, although selectivity has yet to be tested with the 10 and 20 mg/kg SUV dose, it is unlikely that, in the present study, off-target effects are solely responsible for the SUV effects. Moreover, it was reported that in healthy men, acute oral administration of SUV (10–100 mg) produced maximal plasma concentrations within 3 h (Sun et al., 2013) and in rats, a 10-mg oral dose of SUV yielded maximal plasma concentrations 30 min after administration (Cox et al., 2010). Additionally, administration of 30 mg/kg SUV reduced active wake and increased sleep within the first 2 h post-administration in drug-naive rats (Cox et al., 2010; Winrow et al., 2011) suggesting that, even though a history of oxycodone exposure may alter SUV ability to elicit sedative effects, the effects of SUV measured in the present study matched closely to the SUV pharmacokinetics described in earlier preclinical and clinical characterization of SUV. Further, although SUV affinity to bind to OX_1_ and OX_2_ receptors are similar between humans and rats (Cox et al., 2010; Winrow et al., 2011), the time to reach maximal SUV plasma concentrations is longer in humans compared to rats (Cox et al., 2010; Sun et al., 2013). Suvorexant may therefore effectively reduce drug intake and drug seeking in humans for a longer period than what was observed in rats. This will need to be tested carefully in a clinical setting before any decision should be made on safely repurposing SUV for the treatment of prescription OUD.

In conclusion, the results of the present study support continued research on the efficacy of DORAs for the treatment of substance use disorders and indicate that SUV, a drug that is already FDA-approved for the treatment of insomnia, reduces oxycodone self-administration and conditioned reinstatement in both male and female rats. Although the specific OX circuits through which SUV reduces oxycodone taking and seeking are still to be determined, the present findings underscore the significance of targeting the OX system for the treatment of substance use disorders. Studies to determine the efficacy of SUV in clinical populations relative to existing treatments for OUD are warranted. Importantly, the present study suggests that repurposing SUV could be a good alternative for the treatment of prescription OUD to prevent excessive oxycodone taking, craving, and relapse.

## Data Availability

The raw data supporting the conclusion of this article will be made available by the authors, without undue reservation.

## Abbreviations

CPP, conditioned place preferences; DORA, dual orexin receptor antagonist; i.v., intravenous; OX, orexin; OX_1_ receptor, orexin-1 receptor; OX_2_ receptor, orexin-2 receptor; OX receptor, orexin receptor; OUD, opioid use disorder; p.o., oral; RMANOVA, repeated-measures analysis of variance; S^D^, contextual/discriminative stimulus; S^N^, neutral stimulus.
